# Evaluating a decade of post-baccalaureate medical education for international students

**DOI:** 10.5116/ijme.67ce.e765

**Published:** 2025-04-17

**Authors:** Hsiang-Chin Hsu, Tzu-Ching Sung

**Affiliations:** 1 Department of Emergency Medicine, National Cheng Kung University Hospital, College of Medicine, National Cheng Kung University, Tainan, Taiwan; 2 School of Medicine for International Students, College of Medicine, I-Shou University, Kaohsiung, Taiwan

**Keywords:** Post-baccalaureate medical education, Sustainable healthcare development, Global health, Sustainable Development Goal (SDG), Healthcare inequalities

## Introduction

In 2013, a government-funded post-baccalaureate medical education program in Taiwan was launched to train the next generation of medical professionals from allied countries and young students from Africa, the Caribbean, Central America, and the Pacific. The underlying philosophy of this program is rooted in the idea that increasing the number of well-trained doctors in these regions is a more sustainable and effective method of providing aid than sending short-term medical missions to countries in need. The rationale is straightforward: sustainable healthcare development requires a consistent supply of well-educated medical professionals who can provide long-term care and support in their home countries.

This educational program was not only an ambitious undertaking but also one with the potential to impact global health outcomes significantly. By offering scholarships funded by Taiwan’s Ministry of Foreign Affairs and structured under the careful guidance of the International Cooperation and Development Fund, this initiative aimed to cultivate a new generation of doctors who would return to their home countries equipped with the expertise and professionalism needed to enhance local healthcare systems.^1^ These students, hailing from regions where the need for qualified medical personnel is particularly acute, were given the tools to become agents of change in their communities. The program’s intent was clear: to provide a robust educational foundation that would enable these future doctors to make meaningful contributions to their countries' healthcare sectors, thereby reducing health disparities and improving overall health outcomes.

### Evaluating the Program's Effectiveness

The effectiveness of this post-baccalaureate medical training program has been a subject of ongoing evaluation and discussion. Skeptics have often questioned whether these students will become doctors and, more importantly, whether they will return to their home countries after graduation to apply their skills where they are most needed. These concerns, while valid, have largely been addressed through the success stories that have emerged from the program, particularly during the COVID-19 pandemic. The pandemic has underscored the critical gaps in global healthcare and medical education systems, as well as the pressing need for a well-trained healthcare workforce. Graduates of this program, particularly those from the first six batches, have returned to their home countries and played a pivotal role in alleviating the healthcare workload during this unprecedented global health crisis. Their contributions have not only met immediate needs but have also demonstrated the long-term value of the investment made in their education.

### Impact of the COVID-19 Pandemic

The COVID-19 pandemic served as a stark reminder of the importance of having a well-prepared and resilient healthcare workforce. The graduates of this Taiwan-based medical program, who had been trained abroad and then returned to their home countries, were able to step into the breach and provide critical care during the pandemic. This was particularly evident in countries where the healthcare infrastructure was already under strain before the pandemic. The fact that these doctors were able to make such significant contributions speaks volumes about the quality of the education they received and the foresight of the program's architects. It also highlights the importance of long-term, sustainable investment in human capital as a key strategy for improving global health outcomes.

### Key Success Stories

#### St. Lucia

One of the most compelling success stories comes from St. Lucia, where a total of 25 medical students graduated from the program between 2013 and 2018. Upon their return to St. Lucia, these graduates began their practical training as physicians and quickly made a positive impact on the country's healthcare infrastructure and systems, particularly during the 2020 pandemic. Their involvement in community work, health fairs, health drives, home visits, school visits, and other outreach activities exemplifies the program's emphasis on humanitarian dedication and holistic medical care.^2^ This community-focused approach has been instrumental in addressing the healthcare needs of St. Lucia, especially during a time of crisis. Notably, three of these graduates went on to establish a medical clinic, a facility that fully embodies the principles of compassionate and comprehensive care that were emphasized during their training in Taiwan.

#### Eswatini

The impact of the program has not been limited to St. Lucia. A graduate from Eswatini has also made significant contributions to his country's healthcare system since returning home. As a doctor, he has worked at several medical services and has been deeply involved in research on major diseases such as HIV/AIDS, tuberculosis, and malaria. His work has not gone unnoticed; he was recently appointed as the Secretary of the Country Coordinating Mechanism (CCM) organized by the Eswatini Global Fund.^3^ In this role, he assists in the allocation of resources to combat these diseases and oversees the implementation process to ensure that medical care is utilized effectively. His leadership and dedication exemplify the kind of impact that the program's graduates can have on their home countries. By taking on such a pivotal role, he has contributed to the broader international healthcare community and has helped to ensure that Eswatini's healthcare system is better equipped to meet the challenges it faces.

#### Guatemala

Another graduate, this time from Guatemala, has also made a name for himself in the medical field. During his study period from 2013 to 2017, he won a research prize, an achievement that not only highlighted his academic excellence but also served as an inspiration for other young students in Guatemala.^4^ His story has been widely reported, with many people noting how his success abroad has motivated others to seek similar opportunities for cross-country education. His efforts to improve the quality of life in Guatemala, particularly through the management of an integrative medicine clinic in his hometown, have had a lasting impact.^5^ Moreover, his work on the frontlines of COVID-19 prevention during the pandemic has been instrumental in helping to control the spread of the virus in his community. This graduate's achievements underscore the potential of international education programs to produce leaders who can effect positive change in their home countries.

### Global Healthcare Implications

The post-baccalaureate medical education program discussed here is more than just a training ground for future doctors; it is a model for how medical education and humanitarian assistance can be intertwined to create lasting, positive outcomes.^6^ The program's focus on cultivating empathetic and dedicated doctors who are committed to improving holistic medicine in their home countries is not only commendable but also essential in a world where healthcare disparities remain a significant challenge. By investing in the education of students from developing countries, the program is helping to build a global network of medical professionals who are equipped to address the unique healthcare needs of their communities.

The program's success is evident in the stories of its graduates, many of whom have returned to their home countries and made significant contributions to their local healthcare systems. These Schweitzer doctors, as they are often called, have dedicated themselves to serving some of the most vulnerable populations in their countries, often in rural areas where access to medical care is limited. Their work is helping to overturn the medical dilemmas faced by these communities and is contributing to the broader goal of reducing healthcare inequalities. The program’s alignment with Sustainable Development Goal (SDG) 10, which aims to reduce inequalities within and between countries, further underscores its importance as a tool for global health improvement.^7^

### Challenges Faced by Graduates Upon Return

Despite the high-quality training they receive, graduates of Taiwan’s post-baccalaureate medical education program face several challenges upon returning to their home countries. One of the most significant obstacles is the lack of sufficient healthcare infrastructure in many of these regions, which can hinder their ability to fully apply the knowledge and skills they acquired during their training. Limited access to medical resources, outdated technology, and insufficient funding often create barriers to providing high-quality care. Additionally, cultural and systemic healthcare differences can pose challenges, as graduates must navigate local practices and regulatory environments that may not align with the standards they were trained under. Furthermore, the expectation to address complex public health issues, often in underserved or rural areas, places an immense responsibility on these graduates, who may find it challenging to make a substantial impact without adequate support. These challenges underscore the need for continuous support and collaboration between global health programs and local healthcare systems to ensure that the benefits of international medical education are fully realized.

### Enhancing Program Flexibility

As the program continues to evolve, it will be important to build on the successes of the past and address any challenges that may arise. One of the key lessons learned from the COVID-19 pandemic is the need for flexibility and adaptability in medical education. The ability to quickly pivot in response to changing circumstances, such as the emergence of a global health crisis, is critical. The program discussed here has demonstrated this flexibility, particularly in how its graduates have been able to respond to the pandemic. Going forward, there may be opportunities to further enhance the program by incorporating lessons learned from the pandemic and by continuing to focus on the needs of the countries that the program serves.

### Sustaining Long-Term Success

The long-term success of this program will also depend on continued support from both governmental and international organizations. As the world becomes increasingly interconnected, the importance of global health initiatives like this one cannot be overstated. By providing students from developing countries with the education and training they need to become effective medical professionals, the program is helping to build a more equitable and sustainable global healthcare system. This, in turn, contributes to the broader goal of achieving health for all, as outlined in the World Health Organization’s mission.

Taiwan's post-baccalaureate medical education program for international students trains exceptional physicians and nurtures their talents to address healthcare needs in developing countries. The program’s alignment with the values of sustainability and social responsibility sets a remarkable example for other medical education programs worldwide. Graduates are expected to make significant contributions to their countries' medical workforce, particularly in underserved areas, thereby reducing healthcare inequalities and advancing global public health and wellbeing. This program serves as a model for integrating sustainability and social responsibility into medical education, ensuring that future healthcare professionals are not only skilled clinicians but also conscientious global citizens committed to making a positive impact on the world.

### Lessons Learned

Taiwan's post-baccalaureate medical education program for international students offers valuable lessons that can inform similar global initiatives. This success serves as a model for other programs to incorporate cross-cultural and interdisciplinary teaching into their curricula. International literature, including the World Health Organization (WHO) workforce recommendations, emphasizes the importance of interprofessional collaboration and the need to address cultural resistance when adapting medical education innovations across borders.^8^ These insights highlight the broader significance of Taiwan’s program in shaping sustainable healthcare solutions globally and guide other countries seeking to replicate or adapt similar educational frameworks.

It is also essential to contextualize these experiences within the global healthcare landscape. As the WHO has emphasized, IPE is critical for fostering a collaborative and adaptable healthcare workforce.^8^ However, challenges such as professional silos and cultural resistance can hinder the effective implementation of an IPE. The lessons learned from Taiwan’s program, particularly in overcoming cross-cultural barriers and facilitating foreign language instruction, can guide similar programs worldwide. These insights will support other nations in integrating sustainable healthcare education models, ensuring that future graduates are equipped to bridge cultural gaps and address healthcare disparities within their respective regions.

### Acknowledgements 

We extend our sincere gratitude to Ms. Shirley Chou, ICDF Manager, and Assistant Ms. Abby Chen for their dedicated support in assisting international students with daily life, administrative coordination, and general affairs. We also wish to express our appreciation to the former director, Dr. San-Nan Yang, and the current director, Dr. Yung-Chieh Yen, for their exceptional leadership. Their contributions have been invaluable to the success of this work.

### Conflicts of Interest

The authors declare that they have no conflict of interest.

## References

[1] Her K. Doctors in training. Taiwan Today, March 1, 2018. [Cited 18 April 2025]; Available from: https://taiwantoday.tw/Politics/Taiwan-Review/129768/index.

[2] Sonson R. Doctor Nola Rene. Our tropical living. 2022. [Cited 18 April 2025]; Available from: https://ourtropicalliving.com/doctor-nola-rene/.

[3] Gama T. Dr Advocate Dlamini gets CCM top job. Times of Eswatini. 2022. [Cited 18 April 2025]; Available from: https://www.pressreader.com/eswatini/times-of-eswatini/20220423/281638193761380?srsltid=AfmBOoquBrQ8B46qMxvg_ad6W4R96JQiKIqkmPmXRiIldSQlN5cIFNIp.

[4] García PÓ. Beneficiados con becas en Taiwán cuentan su experiencia e invitan a participar en estos programas. Prensa Libre. 2022.

[5] ACUMEDIC Clinica Médica Dr. Pablo Roberto Elías Ruiz. Guatemala 2022. [Cited 5 December 2022]; Available from: https://educ.cl/dr-pablo-roberto-elias-ruiz-12538626018045493173/.

[6] Sinclair S, Kondejewski J, Jaggi P, Dennett L, Roze des Ordons AL, Hack TF (2021). What is the state of compassion education? a systematic review of compassion training in health care.. Acad Med.

[7] Kruk ME, Gage AD, Arsenault C, Jordan K, Leslie HH, Roder-DeWan S, Adeyi O, Barker P, Daelmans B, Doubova SV, English M, García-Elorrio E, Guanais F, Gureje O, Hirschhorn LR, Jiang L, Kelley E, Lemango ET, Liljestrand J, Malata A, Marchant T, Matsoso MP, Meara JG, Mohanan M, Ndiaye Y, Norheim OF, Reddy KS, Rowe AK, Salomon JA, Thapa G, Twum-Danso NAY, Pate M (2018). High-quality health systems in the Sustainable Development Goals era: time for a revolution.. Lancet Glob Health.

[8] WHO. Framework for action on interprofessional education & collaborative practice. 2010 [Cited 22 October 2024]; Available from: https://iris.who.int/bitstream/handle/10665/70185/WHO_HRH_HPN_10.3_eng.pdf?sequence=1. 21174039

